# Editorial: Multi-modal approaches to assess the impact of orthopaedic disease on lower extremity joint function and health

**DOI:** 10.3389/fspor.2024.1389519

**Published:** 2024-03-05

**Authors:** Malek Adouni, Yosra Cherni, Amir Esrafilian, Michael A. Samaan

**Affiliations:** ^1^ Biomedical and Instrumentation Engineering, Abdullah Al Salem University, Khalidiya, Kuwait; ^2^ School of Kinesiology and Exercise Sciences, Faculty of Medicine, Universite de Montreal, Montreal, QC, Canada; ^3^ Department of Applied Physics, University of Eastern Finland, Kuopio, Finland; ^4^ Department of Kinesiology and Health Promotion, University of Kentucky, Lexington, KY, United States

**Keywords:** biomechanics, imaging, orthopaedics, computational modeling, gait

**Editorial on the Research Topic** Multi-modal approaches to assess the impact of orthopaedic disease on lower extremity joint function and health

The number and diversity of methodologies utilized within the field of biomechanics have increased significantly over the past 2–3 decades. This Research Topic sought to establish a collection of manuscripts that utilized various methodologies to assess the impact of orthopedic disease and musculoskeletal conditions on lower extremity joint function and health. The specific conditions addressed within this Research Topic include lower extremity fractures, cerebral palsy, athletic performance/injury prevention, patellofemoral joint mechanics, exoskeletons for rehabilitation, falls, and flatfoot deformity.

Two of the papers within this Research Topic provided insight into lateral malleolar fractures and distal femoral fractures by integrating imaging-based techniques to develop more effective surgical interventions. The first fracture-related paper by Wang et al., delves into the intricate details of Type B lateral malleolar fractures. By employing a three-dimensional perspective, it provides a novel understanding of fracture apexes, which is crucial for developing more effective surgical treatments. This research not only contributes to the field of orthopedic surgery but also offers practical insights for clinicians dealing with such fractures. The second fracture-related paper by Chen et al., focused on identifying fracture lines and comminution zones in AO/OTA types 33A and 33C distal femoral fractures using three-dimensional computed tomography mapping. The distinct features observed in these fractures have significant implications for surgical approaches and internal fixation strategies. The findings, particularly the involvement of the medial femoral epiphysis in 33C fractures, provide valuable insights for selecting surgical approaches, optimizing internal fixation strategies, and guiding the placement of plates or bridging fixation for stabilizing the medial column. These results serve as essential guidelines for both surgical planning and biomechanical studies.

A narrative review article by Keles and Ates focused on the biomechanics of muscle-tendon units in the knee and ankle joints in the context of cerebral palsy. This comprehensive review shed light on the challenges in measuring and modeling muscle forces in people with cerebral palsy. The article also emphasizes the potential for advancements in clinical management and rehabilitation strategies for individuals with cerebral palsy. By bringing attention to these issues, the article emphasizes the potential for advancements in clinical management and rehabilitation strategies for individuals with cerebral palsy, an understudied patient population in the field of biomechanics.

The next article by Chang et al., investigated the application of cutting-edge technology in sports science. By employing inertial measurement units driven by deep learning algorithms to clarify the stages of runner fatigue, hence it opens new avenues for research into athletic performance and injury prevention. This approach not only enhances our understanding of biomechanics and physiology in sports but also it shows how technological innovations can be connected in the field of orthopedic health.

Advances in motion capture and musculoskeletal modeling have played a crucial role in the development and application of robotic technologies for rehabilitation. Lower limb exoskeletons are designed to assist human movement and enhance physiological performance. However, performance and control stability are affected by some model parameters and control algorithms. A manuscript by Gao et al., sought to utilize numerical optimization to adjust the stiffness and damping parameters of a rehabilitation robot to more optimally and accurately track the gait profile in the human-robot interaction.

The evaluation of patellofemoral joint (PFJ) stress is important in understanding the mechanism of PFJ pain. Despite the obvious clinical need to assess PFJ stress, the article by Wang et al., highlights the absence of a definitive “gold standard” for assessment and ongoing efforts to enhance the accuracy of PFJ stress estimations. This article has identified and categorized the various methods used to estimate PFJ stress, the absence of a standardized assessment and the significance of precise evaluation in designing tissue-engineered constructs and evaluating patient recovery. The proposed evaluation scheme prompts future studies to focus on establishing modeling-based platforms for precise PFJ stress calculations, contributing to a deeper understanding of the mechanisms of PFJ pain and optimizing PFJ treatment programs.

Falls are a major concern in the population as they are a leading cause of mortality. The ability to recover from a trip and regain balance, in order to prevent risk of falling, is not well understood. The paper by Namayeshi et al., utilized computational modeling to evaluate the role of the plantar flexor muscle in trip recovery during walking. This study demonstrated the significance of the ankle plantar flexor musculature in successful recovery after tripping during walking and suggests that plantar flexor muscle weakness is an important factor in the risk of tripping during walking.

Flatfoot can lead to instability and poor quality of life and maybe more detrimental in overweight individuals due to increasing plantar loading. The article by Casado-Hernandez et al., sought out to investigate lower extremity mechanics in overweight adults with bilateral flatfoot and healthy-weight adults without flatfoot. The overweight individuals exhibited altered lateral load symmetry during the initial contact and flatfoot phases of the gait cycle, suggesting higher instability in overweight adults with flatfoot compared to healthy-weight adults without flatfoot.

Together, all the articles within this Research Topic represent a multifaceted exploration of orthopedic health. These articles not only reflect the interdisciplinary nature of current orthopedic research but also underline the capability for integrating various scientific, technological and computational approaches to enhance our management of orthopedic diseases. This Research Topic thus serves as a valuable resource for researchers, clinicians, and practitioners in the field, offering insights into the complexities of joint function and health in orthopedic conditions.

